# Research Progress on the Isolation, Purification, Structural Characteristics and Biological Activity Mechanism of *Pleurotus citrinopileatus* Polysaccharides

**DOI:** 10.3390/molecules30132816

**Published:** 2025-06-30

**Authors:** Zixu Liu, Honglei Wang

**Affiliations:** Yantai Institute of China Agricultural University, Yantai 264670, China; zixu_liu@cau.edu.cn

**Keywords:** *Pleurotus citrinopileatus*, polysaccharides, structural characteristics, biological activity, mechanism of action

## Abstract

*Pleurotus citrinopileatus*, a valuable edible fungus characterized by its distinctive light yellow coloration and saprophytic growth on elm wood, has garnered increasing scientific interest due to its diverse bioactive constituents. Among these, polysaccharides derived from *P. citrinopileatus* (PCPs) have received the most extensive research attention. This review summarizes recent advances in the chemical structure and biological activities of PCPs. Structurally, PCPs are primarily composed of repeating units such as →3)-α-D-Glc*p*-(1→ and →6)-α-D-Gal*p*-(1→. Functionally, PCPs exhibit a range of bioactivities, including immunomodulatory, hypoglycemic, and antitumor effects. Furthermore, the underlying mechanisms associated with these biological activities are also explored. This review aims to provide a comprehensive reference for future studies and facilitate the development and application of PCPs as potential functional food ingredients or therapeutic agents.

## 1. Introduction

**Figure 1:**
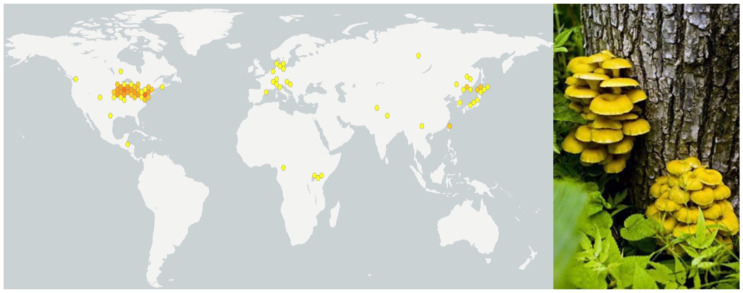
The major distribution of *P. citrinipileatus* in the world [8], and the fruiting body of *P. citrinipileatus* [9]. In the left picture, the dots of different colors represent the richness of *P. citrinipileatus* in this area. The darker the color, the higher the richness.

*Pleurotus citrinopileatus*, named for its light yellow color and its ability to grow on decaying elm wood (Figure 1), belongs to the genus *Pleurotus* and is recognized as a valuable edible fungus [1]. Due to its unique nutritional and medicinal properties, *P. citrinopileatus* has attracted considerable scientific interest in recent years for its rich nutrient content and diverse potential medicinal properties in scientific research and applications. As a medicinal fungus, *P. citrinopileatus* has been widely used in traditional Asian medicine, while modern studies have highlighted its potential applications in the development of functional foods and pharmaceutical products.

Polysaccharides, a complex class of carbohydrates that exhibit biological activity, are composed of ten or more monosaccharide molecules linked by glycosidic bonds. In fungi, polysaccharides serve dual roles: they function both as structural components of the cell wall and as bioactive compounds with significant pharmacological potential. They have been shown to exhibit diverse biological activities, including antioxidant [2], immunoregulatory [3], and antitumor effects [4]. The biological activities of polysaccharides from various edible mushrooms have been extensively studied and widely applied, which has established this field as a major focus of global research. For example, *Ganoderma lucidum* polysaccharides are reported to possess unique antitumor, immunomodulatory, hypoglycemic, lipid-lowering, and antioxidant properties due to their structural characteristics [5]. They have also been found to improve intestinal barrier function and the diversity of the intestinal microbiota.

Significant progress has been made in recent years in the extraction and structural analysis of *P. citrinopileatus* polysaccharides (PCPs), revealing activities such as anti-tumor and immunomodulatory effects. At present, there are in-depth studies on the immune regulatory activity of PCPs [6]. This article studied the active compounds present in PCP composition and analyzed their mechanism of action on human cellular immune regulation based on this. At the same time, there have also been studies on the bioactive components of PCPs, which have been proven to possess anti-tumor activity [7].

Despite these advances, several gaps remain in the research on PCPs. Key challenges include optimizing extraction and purification methods, systematically characterizing structural features, and elucidating the relationships between the chemical structure and biological activities of PCPs. This review aims to systematically summarize the latest research progress on the extraction, purification, structural characteristics, and biological activity mechanisms of PCPs, providing a reference for further research in this field.

## 2. Preparation Technologies of PCPs

### 2.1. Extraction Technologies

#### 2.1.1. Traditional Extraction Method

Hot water extraction (HWE) is one of the most commonly used methods for extracting polysaccharides from edible mushrooms. According to research by Sermwittayawong, D et al., this method is widely used for extracting polysaccharides from gray oyster mushrooms (*Pleurotus ostreatus*) [10]. The method relies on high-temperature heating to disrupt the cell wall, release polysaccharides from the cells, and dissolve them in water. The extraction temperature and extraction time are critical parameters influencing the efficiency of HWE. The advantages of this method include its simplicity and low operational cost. However, a potential disadvantage is the risk of degrading the biological activity of polysaccharides, especially at higher temperatures.

Alkaline extraction is another widely used traditional method, known for its greater extraction efficiency compared to HWE [11]. The principle behind alkaline extraction is the use of high pH to disrupt the interactions between polysaccharides, proteins, polyphenols, and other cell wall components, releasing polysaccharides into the solution. However, the strongly alkaline conditions may lead to degradation or structural alterations in polysaccharides, potentially affecting their biological activity. The general steps involve drying and crushing the material, conducting the extraction under controlled temperature conditions (room temperature to 80 °C), neutralizing the solution after extraction, and precipitating the polysaccharides through acid treatment [12].

In conclusion, both of these extraction methods possess distinct advantages and disadvantages. The choice of extraction technique should be based on specific experimental goals.

#### 2.1.2. Modern Extraction Method

Enzymatic hydrolysis is a widely used modern method for polysaccharide extraction that employs specific enzymes to degrade the cell walls of mushrooms. Currently, this method has been applied to the extraction of β-glucan from *Ganoderma lucidum* [13]. Compared with the above two methods, enzymatic extraction offers high specificity and superior extraction efficiency. Moreover, due to the mild operational conditions of enzymes, it can maintain the biological activity of polysaccharides to a certain extent. However, this method is limited by high cost and operational complexity.

Microwave-assisted extraction (MAE) is an extraction technique that utilizes microwave radiation to generate heat and is widely employed for the extraction of polysaccharides from edible mushrooms. The technique works by rapidly vibrating water molecules within the sample through microwave radiation, thereby producing heat, facilitating cell wall rupture, and promoting the release of bioactive compounds. Studies have shown that compared to HWE, MAE does not significantly alter the yield of polysaccharides [14]. Further studies have demonstrated that the polysaccharides extracted by MAE had better antioxidant activity than those extracted by HWE, thus providing theoretical support for further research on polysaccharide antioxidant mechanisms [14]. MAE offers strong selectivity, reduces impurity extraction, shortens extraction time, and lowers energy consumption to some extent. However, this method may also result in the loss of some thermosensitive components due to elevated temperatures.

Ultrasonic-assisted extraction (UAE) is another modern method that utilizes ultrasonic energy to improve extraction efficiency. The method is primarily based on the cavitation effect generated during ultrasound propagation through liquid media. Specifically, the high-frequency vibration of ultrasound induces the formation of microbubbles in the liquid, which rapidly expand and collapse under pressure changes, generating localized high temperatures and pressures. This effect damages the cell walls and promotes solvent penetration, increasing the release of active ingredients. Studies have shown that UAE can improve the yield of polysaccharides to a certain extent, but it also reduces the antioxidant activity of polysaccharides [15]. This might be due to the fact that UAE alters the structure of the polysaccharides. UAE can significantly shorten the extraction time and improve the polysaccharide extraction rate. However, as mentioned earlier, this method may also affect the activity of polysaccharides to a certain extent, and the equipment cost is relatively high. These factors should be taken into account when using it.

#### 2.1.3. Extraction of *P. citrinopileatus* Polysaccharides

Yang et al. conducted experiments using HWE, alkaline extraction, and UAE to extract polysaccharides from *P. citrinipileatus* and explored the relationship between extraction methods and the structural composition and biological activity of the polysaccharides [16]. Their findings revealed that polysaccharides extracted by UAE yielded the highest amounts and contained higher protein content, while those extracted by HWE had higher sugar and uronic acid levels. Further analysis of the polysaccharides obtained by the three methods revealed that the polysaccharides extracted by HWE and UAE from *P. citrinipileatus* had stronger free radical scavenging ability. Chen et al. conducted an experimental study on the effect of extraction temperature on the biological activity of *Ulmus ostreatus* extract [17]. Studies have shown that polysaccharides extracted at lower temperatures have stronger antioxidant activity and a stronger inhibitory effect on starch-digesting enzymes and angiotensin-converting enzyme (ACE), which may be related to the retention of more bioactive components [17]. The research on *Pleurotus porrigens* by Yim et al. also supports this point [18]. The article indicates that high temperatures exceeding a certain range can also lead to a decrease in the antioxidant activity and total phenol content of *Pleurotus porrigens* [18]. This further demonstrates the importance of controlling the extraction temperature for polysaccharide extraction.

### 2.2. Separation and Purification Technologies

#### 2.2.1. Commonly Used Separation Method

Ethanol precipitation is a widely applied technique for separating crude polysaccharides. This method relies on ethanol’s strong dehydrating properties, which reduce the dielectric constant of polysaccharide solutions, thereby decreasing electrostatic repulsion and promoting the aggregation and precipitation of polysaccharide molecules. Polysaccharides of different molecular weights and other impurities have varying solubility in ethanol, which can be utilized for separation and purification. The ethanol concentration and its addition rate significantly influence the efficiency of precipitation and should be optimized according to the specific physicochemical properties of the target polysaccharides. Xu et al.’s study found that the chemical diversity of polysaccharides, including different structural features and molecular weights, plays a critical role in ethanol-induced polysaccharide precipitation [19]. They demonstrated through experiments that for specific types of pectin, lower molecular weights required higher ethanol concentrations for complete precipitation. When the molecular weight increased from 1 kDa to 270 kDa, the precipitation yields in 80% ethanol increased from 10% to 100%. After standing for a period, the polysaccharide precipitates are collected by centrifugation or filtration. The precipitate is then washed with a low-concentration ethanol solution or another suitable solvent to remove residual impurities and finally dried to obtain purified polysaccharides. This method is simple to implement, cost-effective, and suitable for large-scale preparation. However, it has some limitations, including the potential loss of polysaccharides and difficulty in removing impurities with similar molecular weights, as well as the possible introduction of ethanol residues.

Dialysis is also a commonly used polysaccharide separation method, which utilizes the selective permeability of semipermeable membranes to separate small molecular weight impurities from polysaccharide solutions. Due to their large molecular weight, polysaccharide molecules cannot pass through semipermeable membranes, while small molecule impurities can diffuse into the external solution through semipermeable membranes. The process involves placing the crude extract containing the target polysaccharide into a dialysis bag, which is then immersed in a large volume of buffer solution. The buffer solution is regularly replaced to maintain the concentration gradient and promote the diffusion of impurities. The dialysis time varies depending on the molecular weight and type of impurities, typically lasting several days. After dialysis, the polysaccharide solution is collected from the dialysis bag. The advantages of this method are operational simplicity, minimal damage to polysaccharides, and the ability to remove impurities such as small molecular weight salts and monosaccharides. The disadvantages are low purification efficiency, long dialysis time, and difficulty in removing macromolecular impurities of similar size. There is also a risk of polysaccharide loss, particularly under large concentration gradients.

Ion exchange chromatography is also a widely used and efficient separation method that utilizes the difference in electrostatic interactions between charged polysaccharide molecules and ion exchange resins for separation. Ion exchange resin is composed of small, insoluble particles with fixed charges that can bind with ions in solution with opposite charges. Polysaccharide molecules possess functional groups, such as carboxyl and amino groups, that carry specific charges, enabling ion exchange with ion-exchange resins. The binding affinity between polysaccharide molecules with different charges and resins varies, allowing the elution sequence to be regulated by adjusting the pH and ionic strength of the buffer solution to achieve effective separation and purification. This method has high separation efficiency and can separate polysaccharide molecules with minimal charge differences, while effectively removing impurities with opposite charges. However, it may damage polysaccharide structures, particularly under strong acidic or alkaline conditions. Additionally, the resin requires periodic regeneration, increasing operational complexity. Moreover, some polysaccharides may lack charge and thus cannot be purified using this method. Paulsen et al. used this purification method in their study of lichen-derived polysaccharides [20].

#### 2.2.2. Classical Purification Method

Gel filtration chromatography is a widely used method for the purification of polysaccharides. The column is packed with porous gel beads of varying pore sizes. When the sample solution passes through the chromatographic column, polysaccharide molecules penetrate the gel matrix to different extents depending on their molecular weight. Polysaccharides with larger molecular weights are unable to enter the smaller pores and are eluted earlier. Polysaccharides with smaller molecular weights can enter more pores, so they will be eluted later. The advantages of this method are operational simplicity, minimal structural damage to polysaccharides, effective separation based on molecular weight, and concurrent desalination. However, the method has relatively low resolution and is ineffective for separating polysaccharides with similar molecular weights. Column performance is also highly dependent on gel quality and operating parameters, requiring careful selection of gel type and particle size. Li et al. employed this purification method in their study [21].

Ultrafiltration is also a widely employed technique for the purification of polysaccharides. It is a pressure-driven membrane separation technique that utilizes ultrafiltration membranes with defined pore sizes to separate molecules based on their molecular weights. Due to their large molecular weight, polysaccharide molecules are trapped on one side of the membrane, while small molecule impurities pass through to the other side. This method offers advantages such as high efficiency, rapid processing, concurrent concentration, minimal damage to polysaccharides, and removal of large molecular impurities. However, its disadvantages are high equipment costs, susceptibility to membrane fouling, and the need for regular cleaning and replacement. Xie et al. found in their study on *Cyclocarya paliurus* polysaccharides that ultrafiltration can eliminate low molecular weight polysaccharides, enrich high molecular weight fractions, and significantly improve product quality [22].

#### 2.2.3. Purification of *P. citrinopileatus* Polysaccharides

**Figure 2:**
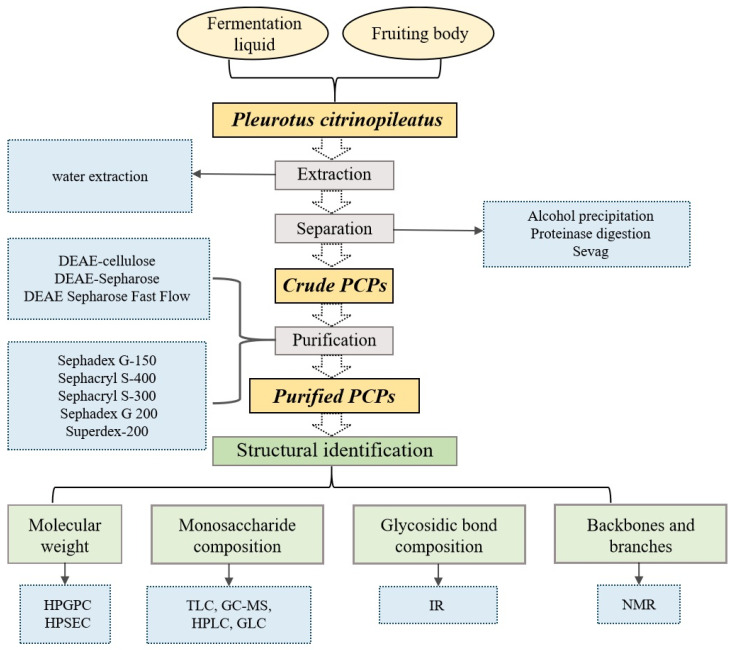
Procedures for extraction, purification, and structural identification of *P. citrinopileatus* polysaccharides.

At present, the purification of *P. citrinopileatus* polysaccharides primarily relies on the aforementioned techniques. Hu et al. employed ultrafiltration in their study on the antihyperglycemic effects of *P. citrinopileatus* polysaccharides, obtaining polysaccharide fractions larger than 100 kDa [23]. In their study, Qian et al. used a 3.5 kDa dialysis bag to remove small molecules and inorganic salts over 24 h, thereby isolating extracellular polysaccharides [24]. K. Minato et al. applied gel filtration chromatography with a Sephacryl S-400 column to isolate bioactive polysaccharides with a molecular weight of approximately 450 kDa (PCPs) [6]. Hao et al. also used gel filtration chromatography in their investigation of the hypoglycemic activity and chemical structure of *P. citrinopileatus* polysaccharides [25]. They dissolved the extracted crude polysaccharide (50 mg) in distilled water (1 mL) and purified it with a packed Sephadex G-150 column. In the experiment, NaCl solution (0–0.5%) was used for elution, and the flow rate was controlled at 0.5 mL/min. A tube was collected every 6 min. Figure 2 below shows the basic procedures for the extraction, purification and structural identification of PCPs. Table 1 below summarizes the methods of polysaccharide extraction, separation and purification used in the current research on PCPs.

## 3. Chemical Characterization of PCPs

Polysaccharides are complex carbohydrates formed by the polymerization of numerous monosaccharide units through glycosidic bonds. Common building blocks include glucose, fructose, and galactose. The way these monosaccharides are linked, specifically the type and position of the glycosidic bonds, such as α-1,4, β-1,4, or α-1,6, plays a crucial role in shaping the structure and properties of the polysaccharide. For instance, α-1,4-glycosidic bonds in starch promote a helical structure, while β-1,4-glycosidic bonds in cellulose result in straight, rigid chains. The introduction of α-1,6-glycosidic bonds creates branch points, as seen in branched starches like amylopectin. These structural variations significantly influence the solubility, viscosity, and overall physical and chemical behavior of polysaccharides. Polysaccharides can exist as linear chains, where monosaccharide units are connected in a straight sequence, or as branched molecules, where side chains extend from the main backbone. The presence of branching increases molecular complexity and steric hindrance, further affecting their functional characteristics. The molecular weight of polysaccharides is typically high, ranging from several thousand to several million Daltons. The molecular weight distribution of polysaccharides is usually not uniform, but rather a range that depends on the degree and mechanism of the polymerization process. The difference in molecular weight can also affect the physical and chemical properties of polysaccharides. These structural characteristics give rise to the diversity and biological functions of polysaccharides, such as energy storage (e.g., starch, glycogen), structural support (e.g., cellulose, chitin), and roles in cell recognition and signal transduction. Research on polysaccharide structure typically primarily focuses on these characteristics to understand their functional properties.

### 3.1. Analysis Methods for Polysaccharide Structure

#### 3.1.1. Chemical Analysis Methods

Chemical analysis of polysaccharides commonly involves acid hydrolysis to break them down into their monosaccharide constituents. These monosaccharides can then be separated and quantitatively analyzed using techniques such as high-performance liquid chromatography (HPLC) or gas chromatography (GC). Since GC typically requires the derivatization of monosaccharides into volatile compounds, HPLC is often preferred for its simplicity. Both HPLC and GC can provide quantitative information on monosaccharide composition, which is relatively simple and fast, and suitable for various types of polysaccharides. In Su et al.’s study, PMP(1-phenyl-3-methyl-5-pyrazolone)-derivatized HPLC was used to determine the composition and proportion of monosaccharides [30]. Vanavil, B. et al. also employed HPLC analysis to determine the purity of extracted polysaccharides in their study on *Sargassum swagzii* polysaccharides [31]. Peng et al. used gas chromatography–mass spectrometry (GC-MS) to analyze black fungus polysaccharides, revealing a neutral sugar composition of Glc/Man = 82.97:12.93. However, acid hydrolysis may lead to the degradation or isomerization of certain monosaccharides, potentially affecting analytical accuracy. Furthermore, chemical methods are limited in that they do not provide detailed information on glycosidic bond linkages, branching structures, or molecular conformation.

#### 3.1.2. Physical Methods

In addition to chemical methods, various physical techniques are commonly employed to analyze polysaccharide structures. For instance, Fourier-transform infrared spectroscopy (FTIR) can detect functional groups in polysaccharides, such as hydroxyl and carbonyl groups, by analyzing the vibrational absorption peaks of molecular bonds within the molecules. FTIR is quick, simple, and does not require complex sample preparation. However, its resolution is low, and it cannot provide detailed structural information, only basic information about functional groups.

Nuclear magnetic resonance (NMR) is a powerful tool for polysaccharide structural analysis. NMR reveals the chemical environment of nuclei within polysaccharide molecules and provides detailed information about monosaccharide types, glycosidic bond configurations, and branching structures. Techniques such as one-dimensional (1D) and two-dimensional (2D) NMR, including COSY, TOCSY, NOESY, HMQC, and HMBC, can deliver comprehensive structural characterization. Zhou et al. used NMR to characterize coconut peel polysaccharides and successfully determined the structure of their main chain [32]. NMR, however, requires high purity and concentration of the sample, and the analysis can be costly and time-consuming. Mass spectrometry (MS), often coupled with chromatography techniques such as LC-MS or GC-MS, is another important analytical method. MS provides molecular weight information and can identify structural fragments of polysaccharides. In the study by Ji et al., GC-MS was used for the methylation analysis of polysaccharides from *Ziziphus jujuba* cv. *Muzao* [33]. While MS can provide molecular weight data and some structural details, the large molecular size of polysaccharides may lead to ionization difficulties, complex spectra, and challenges in data interpretation.

#### 3.1.3. Other Structural Characterization Techniques

X-ray diffraction (XRD) is commonly used to analyze the crystalline structure of polysaccharides, providing information on crystallinity and molecular arrangement. Trabelsi et al. studied the crystal structure of a novel water-soluble polysaccharide from katan seeds using XRD [34]. The principle of XRD involves detecting diffraction patterns generated when X-rays interact with the crystal lattice of the polysaccharide, allowing analysis of crystallinity, unit cell parameters, and molecular packing. The advantage is that it can study the crystallinity, crystal cell parameters, and other information of polysaccharides, which is helpful to understand the macrostructure of polysaccharides. However, it is only applicable to crystalline polysaccharides and provides limited structural detail. It is ineffective for amorphous polysaccharides and does not offer information on glycosidic linkages or branching.

The scanning electron microscope (SEM) is also widely employed in the analysis of polysaccharide structure. SEM works by scanning the sample surface with a focused electron beam, producing secondary electron images that reveal surface topography and microstructure. This method can directly observe the surface morphology of polysaccharides, such as particle size, surface texture, and shape. However, SEM does not provide chemical or molecular structural information—only physical morphology. The sample needs to be pretreated before operation, such as spraying with gold. Wang et al. obtained three polysaccharide fractions from *Citrus aurantium* L. (CAL) by sequential extraction with cold water, hot water, and 1.0 M NaOH, respectively. However, SEM does not provide chemical structural information, only morphological data [35].

#### 3.1.4. Summary

Monosaccharide composition analysis is primarily used to determine the monosaccharide composition and proportion in polysaccharides, while other analytical techniques focus more on detailed structural features. NMR offers the highest resolution and provides the most comprehensive structural information, but it is costly and complex to operate. FTIR is rapid and simple, though it offers limited structural details. MS provides molecular weight and fragment data and is often used in combination with other techniques to enhance structural elucidation. XRD and SEM are mainly used to analyze the physical morphology of polysaccharides. In practice, a combination of multiple analytical methods is often necessary to comprehensively analyze the structure of polysaccharides. For example, the monosaccharide composition is first determined by HPLC or GC, followed by NMR to determine the glycosidic bond connection and branch structure, and finally, the crystalline structure or morphology is analyzed by XRD or SEM. The selection of analytical methods depends on the specific research objectives, the physicochemical properties of the polysaccharides, and the available instrumentation and resources.

### 3.2. Structural Characteristics of P. citrinopileatus Polysaccharides

Currently, the structural analysis of *P. citrinopileatus* polysaccharides often commonly employs multiple complementary analytical techniques. He et al. isolated a water-soluble polysaccharide (PCP60W) from fruiting bodies of *P. citrinopileatus* [1]. The average molecular weight of PCP60W was determined to be 27.4 kDa using high-performance gel permeation chromatography (HPGPC). The polysaccharide PCP60W was described as having a linear structure linked mainly by 1,6-linkages, suggesting a relatively low branching ratio. This structural feature was confirmed through NMR spectroscopy. PCP60W was found to be composed of Gal, 3-O-methyl-Gal, and Glu in a ratio of 3.0:1.0:0.6. The presence of these monosaccharides was determined through monosaccharide composition analysis and methylation analysis. The structure of PCP60W was elucidated using one- and two-dimensional NMR spectroscopy, including COSY, TOCSY, NOESY, HMQC, and HMBC experiments. Methylation analysis was used in combination with NMR spectroscopy to determine the linkage positions and types of sugar units in the polysaccharide. Hao et al. also studied a novel polysaccharide (CMP) from the mycelia of *P. citrinopileatus*, with a molecular weight of 3220 kDa, using HPGPC [26]. CMP was found to consist of D-Man, D-Glu, and D-Gal in a 0.33:1.03:0.21 molar ratio. The backbone of CMP contained →3)-α-D-Glc-(1→ and →3,6)-α-D-Glc-(1→ linkages, while branches included →3)-α-D-Man-(1→ and →3)-β-D-Gal-(1→ linkages. The surface morphology of CMP was described as smooth with an irregular fragmented structure, as observed using SEM or AFM. In Liu et al.’s experiment, the enzymatic extraction residue polysaccharide (ERPS) was extracted from *P. citrinipileatus*, and its antioxidant and hepatoprotective effects were also investigated [36]. The weight-average molecular weight of ERPS was determined to be 130 kDa. The monosaccharide composition included D-Man, L-Rha, D-Glu, D-Gal, D-Xyl, L-Ara, D-GluA, and D-GalA. The composition was analyzed using gas chromatography–mass spectrometry (GC-MS) after hydrolysis and derivatization of the polysaccharide, enabling the identification and quantification of individual monosaccharide units. Structural analysis indicated that ERPS had β-type glycosidic pyranose linkages, confirmed by FTIR and NMR spectroscopy.

Compared with other fungal polysaccharides, such as *Ganoderma lucidum* polysaccharides and *Agaricus bisporus* polysaccharides, *P. citrinopileatus* polysaccharides exhibit distinct structural features that define their unique characteristics. *P. citrinopileatus* polysaccharides have a molecular weight in the megadalton range (approximately 1062 kDa), similar to *A. bisporus* polysaccharides (784 kDa), but the exact molecular weight range for *G. lucidum* polysaccharides varies widely, with some high molecular weight polysaccharides being greater than 300 kDa [37,38,39]. *P. citrinopileatus* polysaccharides have a complex branching structure, while *G. lucidum* polysaccharides adopt a flexible, random linear conformation with specific branching moieties that contribute to their immunomodulatory activity. *P. citrinopileatus* polysaccharides are composed of a mix of D-Galactose, D-Glucose, D-Galacturonic acid, and D-Glucuronic acid, while *G. lucidum* polysaccharides mainly consist of glucose and galactose [26,40]. *A. bisporus* polysaccharides, on the other hand, include a broader spectrum of monosaccharides, such as ribose, rhamnose, arabinose, xylose, mannose, glucose, and galactose [38]. These structural differences, particularly in molecular weight, branching patterns, and monosaccharide composition, play a crucial role in the distinct biological activities of each fungal polysaccharide, contributing to their unique properties and potential applications. Table 2 below summarizes the methods used for polysaccharide structure identification in current studies on PCPs and lists the molecular weights and monosaccharide compositions of several known PCPs, as well as providing schematic structural diagrams.

## 4. Biological Activities of PCPs

### 4.1. Immunomodulation Activity

**Figure 3:**
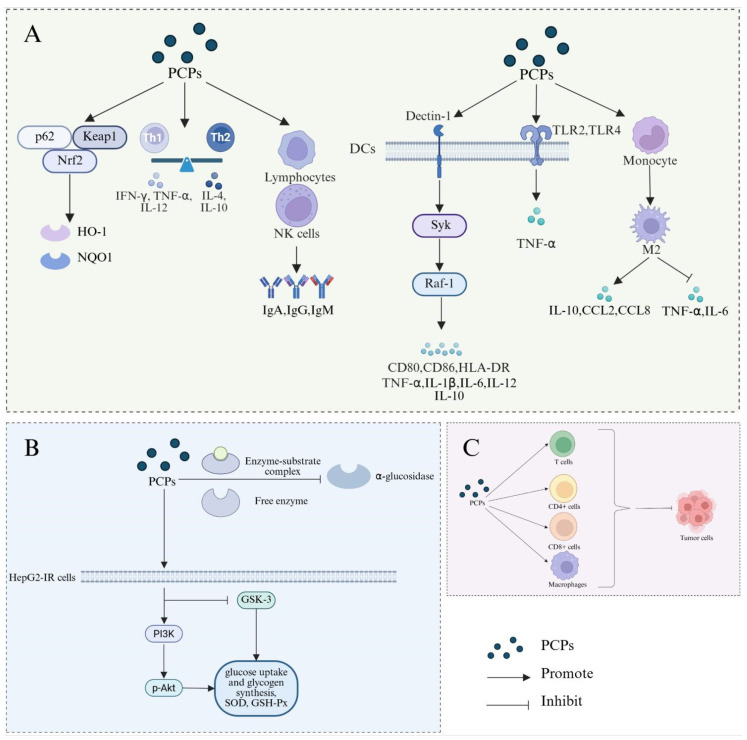
Biological activity mechanism of PCPs. (**A**) The regulatory effect of PCPs on immune activity. (**B**) The hypoglycemic effect of PCPs. (**C**) The anti-tumor activity of PCPs.

Polysaccharides extracted from *P. citrinopileatus* mycelia (CMP) exhibit notable immunomodulatory activity, primarily by restoring immune function in cyclophosphamide-induced immunosuppressed mice (Figure 3A). Mechanistically, CMP activates the p62/Keap1/Nrf2 signaling pathway, leading to the nuclear translocation of Nrf2 and subsequent upregulation of antioxidant enzymes such as HO-1 and NQO1, thereby mitigating oxidative stress-related immune damage [41]. Concurrently, CMP modulates Th1/Th2 balance by suppressing Th1-associated cytokines (IFN-γ, IL-12, TNF-α) while promoting Th2 cytokines (IL-4, IL-10), thereby rectifying immune dysregulation [41]. The polysaccharide also enhances cellular immunity by boosting splenic lymphocyte proliferation and NK cell cytotoxicity, and increasing immunoglobulin levels (IgA, IgG, IgM) in both serum and spleen [41]. This dual regulation of oxidative and immune signaling pathways highlights CMP’s potential as a bioactive agent for immunoprotection and homeostatic regulation under immunosuppressive conditions.

The immunomodulatory mechanism of another polysaccharide, PCPS, extracted from *P. citrinopileatus*, has also been extensively studied (Figure 3A). The immunomodulatory activity of PCPS stems from its multifaceted activation of human dendritic cells (DCs) through receptor-mediated signaling cascades. Structural characterization identifies PCPS as a β-1,3-branched β-1,6-glucan, enabling recognition by Dectin-1, a key fungal β-glucan receptor on DCs [6]. Dectin-1 engagement triggers downstream Syk- and Raf-1-dependent pathways, promoting DC maturation (indicated by the upregulation of CD80, CD86, and HLA-DR) and the secretion of both pro-inflammatory cytokines (TNF, IL-1β, IL-6, IL-12) and the anti-inflammatory cytokine IL-10, with anti-Dectin-1 antibodies markedly suppressing these effects [6]. PCPS further activates Toll-like receptors (TLRs), directly stimulating TLR2 and TLR4, while polymyxin B pretreatment confirms that TLR4 activation is endotoxin-independent [6]. Polymyxin B is commonly used to verify the presence of endotoxins in samples due to its ability to bind and neutralize lipopolysaccharides (LPS), the major components of endotoxins [42]. TLR2 co-stimulation amplifies TNF secretion synergistically, whereas TLR4 activation exerts additive effects, suggesting coordinated signaling between Dectin-1 and TLRs to enhance immune modulation [6]. Beyond DCs, PCPS modulates macrophage polarization, skewing monocytes toward an M2 phenotype characterized by elevated IL-10 secretion and suppressed TNF-α and IL-6, while upregulating anti-inflammatory chemokines CCL2 and CCL8 [43,44]. This polarization is mediated by early-stage Dectin-1 and TLR2 engagement, with laminarin (Dectin-1 antagonist) and anti-TLR2 antibodies reversing TNF suppression and confirming receptor specificity [43]. In murine models of allergic contact dermatitis, oral PCPS administration attenuated inflammation by restoring M2 macrophage populations and rebalancing cytokine dynamics, highlighting its systemic immunoregulatory potential [44]. These mechanisms underscore PCPS’ capacity to harmonize pro- and anti-inflammatory responses through structural specificity for Dectin-1 and TLRs, coupled with downstream NF-κB and MAPK pathway modulation, positioning it as a multifaceted immunomodulator with therapeutic relevance.

In summary, the immunomodulatory mechanisms of CMP and PCPS from *P. citrinopileatus* involve a complex interplay of oxidative stress regulation, cytokine modulation, and immune cell activation. These mechanisms highlight the potential of *P. citrinopileatus* polysaccharides as therapeutic agents for various immune-related disorders, offering a multifaceted approach to immune modulation and homeostasis.

### 4.2. Hypoglycemic Activity

An acid polysaccharide was isolated from the fermented broth of *P. citrinopileatus* (CFP). Just like CMP mentioned earlier, the research on the biological activity of CFP has attracted widespread attention, and the mechanism of their hypoglycemic activity was investigated (Figure 3B). CFP inhibits α-glucosidase via non-competitive inhibition, reducing postprandial glucose absorption by binding to the enzyme/substrate complex. Meanwhile, CFP demonstrated a dose-dependent inhibition of α-glucosidase with an IC50 value of 0.556 mg/mL. This indicates that CFP effectively reduces postprandial hyperglycemia by blocking the breakdown of carbohydrates in the digestive tract. In insulin-resistant HepG2 (HepG2-IR) cells, CFP enhances glucose uptake and glycogen synthesis while alleviating oxidative stress via elevated SOD and GSH-Px activities [25]. This means that CFP not only addresses the immediate issue of hyperglycemia but also helps to restore normal cellular glucose metabolism. Its uronic acid content and triple-helix conformation likely facilitate enzyme interaction and cellular uptake, supporting dual action on enzymatic activity and insulin signaling [25,45,46]. This underscores its potential as a therapeutic agent for diabetes management. CMP demonstrates mixed-type α-glucosidase inhibition, targeting both free enzyme and enzyme/substrate complexes. Mechanistically, CMP activates the PI3K/Akt pathway in HepG2-IR cells, upregulating PI3K expression, promoting Akt phosphorylation (p-Akt), and suppressing GSK-3 activity [26]. The structural characteristics of CMP, such as its backbone composed of →3)-α-D-Glc*p*-(1→ and →3,6)-α-D-Glc*p*-(1→, are also implicated in its hypoglycemic activity [26]. The presence of these specific glycosidic linkages may enhance the binding affinity of CMP to receptors involved in glucose metabolism, thereby optimizing its ability to activate the PI3K/Akt pathway [26]. This structural insight provides a basis for the rational design of future hypoglycemic agents derived from *P. citrinopileatus*.

The antihyperglycemic mechanism of *P. citrinopileatus* polysaccharides (SPPC) involves multifaceted regulation of glucose and lipid metabolism. At 0.4 g/kg body weight, SPPC reduced fasting blood glucose by 44% in STZ-induced diabetic rats, which correlated with attenuated pancreatic islet damage, suggesting β-cell protection through the inhibition of glucolipotoxicity-induced apoptosis [29,47]. Immunomodulatory effects, such as enhanced CD4^+^/CD8^+^ T cell proliferation, may further mitigate immune-mediated β-cell dysfunction [29].

In summary, the polysaccharides derived from *P. citrinopileatus* exhibit a broad spectrum of hypoglycemic activities through diverse mechanisms, including α-glucosidase inhibition, insulin signaling enhancement, and oxidative stress reduction. These findings highlight the potential of *P. citrinopileatus* as a rich source of bioactive compounds for the development of functional foods and pharmaceuticals aimed at diabetes management.

### 4.3. Antitumor Activity

The research on *P. citrinopileatus* polysaccharide (PCP) has shown significant antitumor activity against H22 hepatoma in mice. PCP was extracted and purified from *P. citrinopileatus*, and it exerts antitumor effects by modulating immune cell activity [29]. The results indicated that PCP could effectively suppress H22 solid tumor growth, protect immune organs, and improve inflammation and anemia in tumor-bearing mice [7]. PCP induced apoptosis in H22 hepatoma cells, as evidenced by Annexin V-FITC/PI double staining and JC-1 staining, and arrested the cell cycle at the S phase, as determined by PI staining. The study demonstrated that PCP has potential therapeutic value for liver cancer treatment with low toxicity and strong antitumor activity. Future research should focus on further exploring the structure–activity relationship of PCP to better understand its antitumor mechanisms and optimize its application in cancer therapy.

The antitumor activity of the water-soluble polysaccharide (SPPC) derived from *P. citrinopileatus* has been investigated in several studies (Figure 3C). Wang et al. investigated the optimization of SPPC production and its antitumor effects. Animal experiments showed that the administration of SPPC to mice bearing artificially induced pulmonary metastatic tumors resulted in a significant increase in the number of T cells, CD4^+^ cells, CD8^+^ cells, and macrophages [29]. The proliferation rate of pulmonary sarcoma lesions was significantly reduced. Specifically, daily administration of SPPC at a dose of 50 mg/kg for 12 days led to a significant increase in immune cells compared to untreated tumor-bearing mice. The number of tumor nodules and pulmonary tumor cells decreased in SPPC-treated mice, indicating that SPPC may inhibit the proliferation of pulmonary metastases. The study highlights the potential of SPPC from *P. citrinopileatus* as an antitumor agent with immune-enhancing properties. Future research could focus on further elucidating the structure–activity relationship and the specific mechanisms underlying the antitumor effects of SPPC.

Comparative analysis of PCP and SPPC indicates that both polysaccharides exhibit strong antitumor activity through immune modulation and direct cytotoxic effects. These results suggest that *P. citrinopileatus* polysaccharides may have broad-spectrum antitumor effects applicable to different types of cancers.

In conclusion, the polysaccharides from *P. citrinopileatus* represent promising natural antitumor agents with low toxicity and multiple mechanisms of action. Further research into their structural features will be essential for developing structure-based applications in cancer therapy.

### 4.4. Discussion on the Biological Activity Research of PCPs

At present, most of the research on these activities has remained at the molecular level and there are very few in vivo studies, which limits the further development and utilization of PCPs. Therefore, it is highly necessary to bring the research on PCPs to the in vivo level, and the in vivo research is often carried out relying on the gut microbiota (GM). For example, in aged and tumor-challenged mice, a heteropolysaccharide L2 from *Lentinula edodes* restored immune responsiveness and partially reversed age-related microbiota changes, as shown by high-throughput sequencing of fecal samples [48]. Fermentation of a β-glucan-rich extract (LePS40) in human fecal models produced elevated SCFAs (butyrate, propionate), suppressed inflammation in an intestinal co-culture assay, and selectively enriched beneficial bacterial taxa while depleting those linked to colorectal cancer [49]. In addition, a study found that a polysaccharide derived from the spore of *Ganoderma lucidum*, when combined with Paclitaxel, can act as an adjuvant against Pertussis toxin in breast cancer treatment [50]. This combination restores GM dysbiosis, increasing levels of beneficial bacteria such as *Ruminococcus* and *Bacteroides* while reducing the presence of cancer-risk genera such as *Odoribacter* and *Desulfovibrio* [50]. Meanwhile, recent in vivo studies demonstrated that *Ganoderma lucidum* polysaccharides (GLP) extracted from sporoderm-removed spores significantly alleviate AOM/DSS-induced colitis and colorectal tumorigenesis in mice through restoration of gut microbiota balance, enhanced production of short-chain fatty acids (SCFAs), improved intestinal barrier integrity, and suppression of TLR4/MyD88/NF-κB signaling pathways [51]. Another study showed that GLP enhanced CD8^+^ and Th1 anti-tumor immunity in colorectal cancer-bearing mice, reduced the kynurenine/tryptophan ratio, increased SCFA production, and potentiated the efficacy of anti-PD-1 immunotherapy [52]. These examples illustrate that edible mushroom polysaccharides, including those from shiitake and reishi mushrooms, can exert significant biological activities through modulation of the GM. Future research on the biological activity of PCPs can also refer to the ideas mentioned above and further discuss the mechanism of action of the GM on its biological activity in vivo.

## 5. Discussion

In recent years, extensive research has been conducted on the extraction, separation, purification, chemical structure, and biological activity of polysaccharides. When it comes to industrial-scale production of polysaccharides, cost-effectiveness and efficiency are of utmost importance. The most widely used method for polysaccharide extraction is hot water extraction, which is advantageous for its simplicity and low cost, making it highly suitable for large-scale industrial applications. However, high extraction temperatures may compromise the biological activity of polysaccharides. Therefore, in the context of industrial production, it is crucial to strike a balance between cost and bioactivity. New techniques such as enzymatic hydrolysis or combinations of multiple methods should be explored to enhance extraction yield while preserving bioactivity. These advanced methods, although potentially more expensive initially, may offer long-term benefits by reducing the need for additional purification steps and improving the overall quality of the final product. At present, the most common method for separating polysaccharides from *P. citrinopileatus* is alcohol precipitation, which is often used in combination with other methods such as Sevag and proteinase digestion. This method is simple to operate and cost-saving, making it a popular choice for industrial-scale production. However, it can also cause a certain loss of polysaccharides to a certain extent. To address these challenges, while maintaining cost-effectiveness, other methods such as dialysis and ion-exchange chromatography have gradually been applied to the separation of polysaccharides from edible fungi. These methods can improve the purity and yield of polysaccharides, but their higher operational costs and complexities need to be carefully evaluated in the context of large-scale production. In the future, research on PCPs should consider the adoption of new separation methods to improve efficiency. Techniques like gel filtration chromatography and ultrafiltration are commonly used for purifying polysaccharides and have also been applied to the purification of PCPs. While these methods are highly effective in laboratory settings, their scalability and cost implications for industrial production must be thoroughly assessed. For instance, optimizing ultrafiltration membrane materials and operating conditions could reduce costs while maintaining high purification efficiency. Similarly, integrating advanced techniques with traditional methods in a hybrid approach might offer a cost-effective solution without compromising the quality of the polysaccharides. By carefully considering both technological advances and economic feasibility, the industrial production of PCPs can be significantly enhanced.

In the structural analysis of PCPs, we found that many studies have preliminarily explored the structures of certain polysaccharides derived from *P. citrinopileatus* using methods such as NMR, GC-MS, and FT-IR. Based on existing literature descriptions, we have drawn several simple structural diagrams of PCPs. For instance, one representative homogeneous fraction, CMP, has been characterized by a molecular weight of approximately 3220 kDa and a monosaccharide composition of Man/Glc/Gal = 0.33:1.03:0.21. Its backbone is primarily composed of →3)-α-D-Glc*p*-(1→ and →3,6)-α-D-Glc*p*-(1→ residues, with branching points at →3)-α-D-Man*p*-(1→ and →3)-β-D-Gal*p*-(1→. However, CMP represents only a single fraction of the diverse polysaccharide pool found in *P. citrinopileatus*. Given the complexity and heterogeneity of fungal polysaccharides, each isolated fraction may possess unique structural and biological properties. Therefore, it is crucial to further isolate, purify, and structurally characterize other fractions to obtain a comprehensive understanding of PCPs’ structural diversity. In future research, combining traditional techniques with high-resolution methods such as multi-dimensional NMR, methylation analysis, and mass spectrometry-based sequencing approaches (e.g., MALDI-TOF-MS, LC-MS/MS) may offer more detailed insights into linkage types, substitution positions, and repeating units. Integration with computational tools such as molecular modeling and AI-driven structural prediction could also accelerate the elucidation of structure–function correlations.

Current research has demonstrated that PCPs possess a wide range of biological activities, including immunomodulatory, hypoglycemic, antioxidant, and antitumor effects. PCPs have been shown to activate macrophages, stimulate the secretion of pro-inflammatory cytokines such as IL-6 and TNF-α, and enhance lymphocyte proliferation, indicating a potential role in immune regulation. In metabolic disease models, PCPs have been observed to lower blood glucose levels and improve insulin sensitivity, potentially through the modulation of GM composition and metabolic signaling pathways. In the context of oncology, certain PCP fractions have demonstrated the ability to suppress cancer cell growth, promote apoptosis, and alleviate oxidative stress, further underscoring their therapeutic potential. Despite these promising findings, the structure–activity relationship (SAR) of PCPs remains poorly understood. While this review summarizes the current knowledge and proposes potential mechanistic pathways illustrated with diagrams, the specific structural determinants underpinning these bioactivities have yet to be systematically elucidated. One of the major challenges in SAR studies lies in the heterogeneity of fungal polysaccharides and the methodological inconsistencies in their extraction, purification, and characterization. Consequently, existing data are often fragmented and difficult to compare across studies.

To address this gap, future research should prioritize the isolation and structural characterization of homogeneous PCP fractions, followed by standardized biological evaluations. Correlating structural parameters—such as molecular weight, degree of branching, monosaccharide composition, and glycosidic linkage patterns—with specific bioactivities will be essential. Moreover, the integration of advanced technologies, including multi-dimensional NMR, high-resolution mass spectrometry, and multi-omics approaches such as transcriptomics, metabolomics, and microbiome profiling, may provide more comprehensive insights into the underlying molecular mechanisms. Ultimately, these efforts will contribute to the systematic development of PCPs as functional food ingredients or therapeutic agents, enabling a transition from empirical application to evidence-based design and quality-controlled utilization.

## 6. Conclusions

PCPs exhibit a variety of biological activities, including immunomodulation, hypoglycemic effects, and antitumor activities. This article compares and summarizes the extraction, separation, and purification methods for PCPs and analyzes the advantages and disadvantages of each method. Additionally, this article reviews the chemical structural characteristics and biological activity mechanisms of PCPs, with the aim of providing a theoretical foundation for the further development of PCPs as functional food ingredients and supporting future research in this field.

## Figures and Tables

**Table 1:** Extraction, separation, and purification of *P. citrinopileatus* polysaccharides.

Fraction	Source	Extraction	Separation	Purification	Ref.
CMP	Liquid fermentation	Water extraction (*w*:*v* = 1:20, 75 °C, 2 h, 3 times)	Sevag, alcohol precipitation	Sephadex G-150	[26]
CFP	Liquid fermentation	ND	Sevag, alcohol precipitate *P. citrinopileatus* medium	DEAE-cellulose, Sephadex G-150	[25]
PCPS	ND	Hot water extraction	ND	DEAE-Sepharose, Sephacryl S-400	[6]
PCP	Provided by Garden Edible Fungus Co., Ltd. (Jilin, China)	Water extraction (4 °C, 4 times)	Sevag, alcohol precipitation	Sephadex G-150	[7]
PCP60W	ND	Boiling water extraction (2 h, 3 times)	Alcohol precipitation, anion-exchange, gel chromatography, diethylaminoethyl Sepharose fast flow	Sephacryl S-300	[1]
FP_2_-Pc /FS_2_-Pc	Furnished by Yuki Cogumelos Company	Water extraction (10 °C, 6 h)	EtOH precipitation, strongly acidic cation exchange resin	ND	[27]
PCP-W1	ND	Boiling water extraction (30 L, 6–7 h, 6 times)	EtOH precipitation, proteinase digestion, Sevag	DEAE Sepharose Fast Flow, Sephadex G 200	[9]
PSI/PSII	Preserved at the Plant Protection Institute of the Beijing Academy of Agricultural and Forestry Sciences	Water extraction (*w*:*v* = 1:50, 90 °C, 3 h, 4 times)	Sevag, alcohol precipitation	DEAE-cellulose, Superdex-200	[28]
SPPC	Liquid fermentation	ND	Alcohol precipitate *P. citrinopileatus* medium	ND	[29]

ND means not detected.

**Table 2:** Chemical structure characteristics of *P. citrinopileatus* polysaccharides.

Fraction	Molecular Weight (kDa)	Molar Ratios of Constituent Monosaccharides	Main Chain	Branch Chain	Analysis Technique	Chemical Structure	Ref.
CMP	3220 kDa	Man: Glc: Gal = 0.33:1.03:0.21	→3)-α-D-Glc*p*-(1→ and →3,6)-α-D-Glc*p*-(1→	→3)-α-D-Man*p*-(1→ and →3)-β-D-Gal*p*-(1→	HPGPC, GC-MS, NMR, SEM, FT-IR	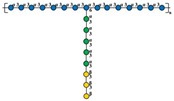	[26]
CFP	1062 kDa	Gal: Glc: GalA: GlcA = 20.53:28.75:5.55:45.17	→3)- α-D-Glc*p*-(1→, →3)-α-D-GlcA*p*-(1→ and →3,6)- α-D-Glc*p*-(1→	→3)-β-D-GalA*p*-(1→and →3)-β-D-Gal*p*-(1 →	HPGPC, FT-IR, NMR, GC-MS, Congo-red analysis, SEM	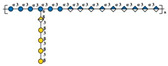	[25]
PCPS	450 kDa	Glc	→6)-β-D-Glc*p*-(1→	→3)-β-D-Glc*p*-(1→	GC-MS, NMR	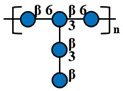	[6]
PCP	730 kDa	Ara: Gal: Glc: Xyl: Man: GlcA = 0.66: 14.59: 10.77: 1: 0.69: 0.23	ND	ND	HPGPC, FT-IR, NMR, SEM, Congo red assay	ND	[7]
PCP60W	27.4 kDa	Gal:3-O-Me-Gal:Glc = 3.0:1.0:0.6	→6)-α-D-Gal*p*-(1→, →6)- α-D-Gal*p*-3-OMe-(1→ and →4)- α-D-Glc*p*-(1→	/	GC-MS, NMR, HPSEC, HPLC, IR	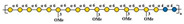	[1]
FP_2_-Pc	37.6 kDa	Gal:3-O-Me-Gal = 2:1	→6)-α-D-Gal*p*-(1→ and →6)- α-D-Gal*p*-3-OMe-(1→	/	GC-MS, HPSEC, NMR	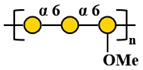	[27]
FS_2_-Pc	28.5kDa	Gal:3-O-Me-Gal = 1:1	→6)-α-D-Gal*p*-(1→ and →6)- α-D-Gal*p*-3-OMe-(1→	/	GC-MS, HPSEC, NMR	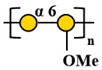	[27]
PCP-W1	45 kDa	Glc	→6)-β-d-Glc*p*-(1→ and → 3,6)-β-d-Glc*p*-(1 →	→1)-β-d-Glc*p*-(3 → 1)-β-d-Glc*p*	HPSEC, HPLC, FTIR, GC, GC–MS, NMR	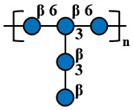	[9]
PSI	1216 kDa	Ara:Man:Glc:Gal = 1:6.2:6.3:67.2	ND	ND		ND	[28]
PSII	16.08 kDa	Xyl:Glc:Gal = 1:83.9:4.2	ND	ND		ND	[28]
SPPC	>100 kDa	Glc:Man = 4.3:1.0	ND	ND		ND	[29]

“ND” means not detected, “/” means that it has already been represented in the main chain.

## Data Availability

No new data were created or analyzed in this study. Data sharing is not applicable to this article.
